# Deciphering genetic signatures by whole exome sequencing in a case of co-prevalence of severe renal hypouricemia and diabetes with impaired insulin secretion

**DOI:** 10.1186/s12881-020-01031-z

**Published:** 2020-05-06

**Authors:** Motohiro Sekiya, Takaaki Matsuda, Yuki Yamamoto, Yasuhisa Furuta, Mariko Ohyama, Yuki Murayama, Yoko Sugano, Yoshinori Ohsaki, Hitoshi Iwasaki, Naoya Yahagi, Shigeru Yatoh, Hiroaki Suzuki, Hitoshi Shimano

**Affiliations:** Department of Internal Medicine (Endocrinology and Metabolism), Faculty of Medicine, University of Tsukuba, 1-1-1 Tennodai, Tsukuba, Ibaraki 305-8575 Japan

**Keywords:** Whole exome analysis, Hypouricemia, *SLC22A12*, *ABCG2*, Impaired insulin secretion, *HNF1A*, *NKX6.1*

## Abstract

**Background:**

Renal hypouricemia (RHUC) is a hereditary disorder where mutations in *SLC22A12* gene and *SLC2A9* gene cause RHUC type 1 (RHUC1) and RHUC type 2 (RHUC2), respectively. These genes regulate renal tubular reabsorption of urates while there exist other genes counterbalancing the net excretion of urates including *ABCG2* and *SLC17A1*. Urate metabolism is tightly interconnected with glucose metabolism, and *SLC2A9* gene may be involved in insulin secretion from pancreatic β-cells. On the other hand, a myriad of genes are responsible for the impaired insulin secretion independently of urate metabolism.

**Case presentation:**

We describe a 67 year-old Japanese man who manifested severe hypouricemia (0.7 mg/dl (3.8–7.0 mg/dl), 41.6 μmol/l (226–416 μmol/l)) and diabetes with impaired insulin secretion. His high urinary fractional excretion of urate (65.5%) and low urinary C-peptide excretion (25.7 μg/day) were compatible with the diagnosis of RHUC and impaired insulin secretion, respectively. Considering the fact that metabolic pathways regulating urates and glucose are closely interconnected, we attempted to delineate the genetic basis of the hypouricemia and the insulin secretion defect observed in this patient using whole exome sequencing. Intriguingly, we found homozygous Trp258* mutations in *SLC22A12* gene causing RHUC1 while concurrent mutations reported to be associated with hyperuricemia were also discovered including *ABCG2* (Gln141Lys) and *SLC17A1* (Thr269Ile). SLC2A9, that also facilitates glucose transport, has been implicated to enhance insulin secretion, however, the non-synonymous mutations found in *SLC2A9* gene of this patient were not dysfunctional variants. Therefore, we embarked on a search for causal mutations for his impaired insulin secretion, resulting in identification of multiple mutations in *HNF1A* gene (MODY3) as well as other genes that play roles in pancreatic β-cells. Among them, the Leu80fs in the homeobox gene *NKX6.1* was an unreported mutation.

**Conclusion:**

We found a case of RHUC1 carrying mutations in *SLC22A12* gene accompanied with compensatory mutations associated with hyperuricemia, representing the first report showing coexistence of the mutations with opposed potential to regulate urate concentrations. On the other hand, independent gene mutations may be responsible for his impaired insulin secretion, which contains novel mutations in key genes in the pancreatic β-cell functions that deserve further scrutiny.

## Background

**Fig. 1:**
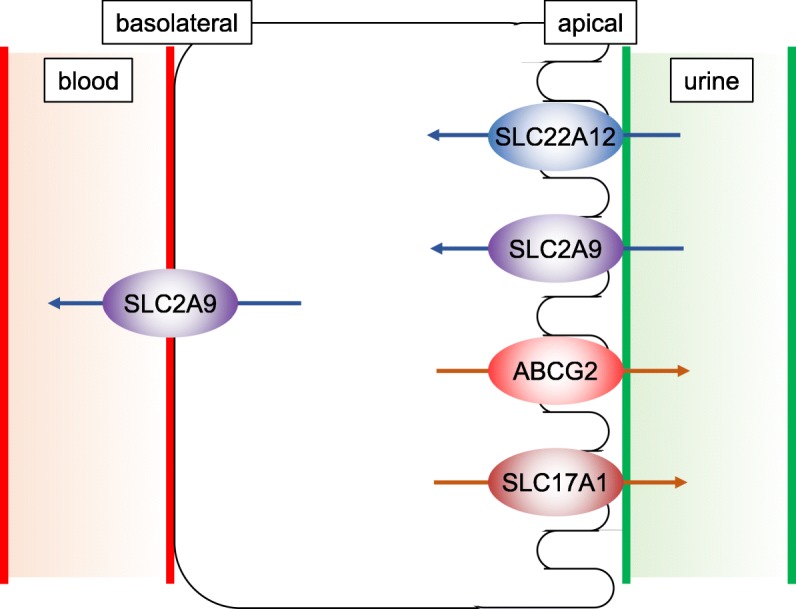
Schematic description of urate transport in renal tubular cells. Arrows indicate the direction of urate transport

The serum urate concentrations are tightly regulated through multiple complex processes including hepatic production and renal excretion as well as intestinal secretion [1, 2]. The renal tubular transport of urates is regulated bidirectionally [3]: The reabsorption is regulated mainly through two major solute carrier (SLC) transporters, SLC22A12 (also known as URAT1, urate anion transporter 1) [4, 5] or SLC2A9 (also known as GLUT9, glucose transporter 9) [6–8] while several transporters for excretion have been identified including ABCG2 (ATP-binding cassette transporter G2) [9, 10], ABCC4 [11], SLC17A1 [12, 13] and OAT (organic anion transporter) family members [11]. The net renal urate excretion is largely determined by the balance of these reabsorption and excretion. The loss of function mutations in *SLC22A12* and *SLC2A9* causes renal hypouricemia (RHUC) type 1 and type 2, respectively [14]. The SLC22A12 is expressed at the apical membrane of proximal tubules while SLC2A9 isoforms are localized to both the apical and basolateral membrane [11] (Fig. 1).

As exemplified by the fact that SLC2A9 facilitates the transport of not only urates but also glucose and fructose, urate metabolism is closely interconnected with glucose metabolism. Indeed, both positive and negative correlations between serum urates and blood glucose have been demonstrated. Insulin resistance increases serum urate levels independently of obesity [15]. Based on this positive correlation, serum urate levels were proposed as a potential predictor of type 2 diabetes occurrence [16]. Despite the accumulating observations supporting this positive correlations, negative correlations have long been well appreciated clinically as well [17, 18]. Renal urate excretion has been known to be competed with glycosuria [19], that might be at least in part explained by the dual transporting properties of SLC2A9 for glucose and urates [20]. Moreover, SLC2A9 has been reported to be expressed in pancreatic β-cells where SLC2A9 was proposed to facilitate the glucose uptake to increase glucose induced insulin secretion [21].

Here we report a case with severe hypouricemia accompanied by diabetes with impaired insulin secretion where whole exome sequencing revealed gene mutations responsible for these metabolic disorders.

## Case presentation

A 67 year-old Japanese man was referred to our hospital for the treatment of diabetes with the concomitant concern about his severe hypouricemia. He was admitted to our hospital and biochemical data were collected under hospitalized conditions. On admission, his body mass index (BMI) was 25.1 (height: 174.1 cm, weight: 76.2 kg), excluding the possible contributions of obesity-induced alteration of urate metabolism. Although the historical onset and progression of his hypouricemia was unclear, he exhibited severe hypouricemia with hyperuricosuria (serum urate levels: 0.7 mg/dl (3.8–7.0 mg/dl) (41.6 μmol/l (226–416 μmol/l)), fractional excretion of urate (FEUA) was 65.5%) without any signs of kidney dysfunction (serum creatinine levels: 0.7 mg/dl, creatinine clearance: 109.1 ml/min, estimated glomerular filtration rate (eGFR): 85.4 ml/min/1.73m^2^). His urinary fractional excretion of urates was elevated but relatively modest compared to the reported cases of severe hypouricemia [8, 22]. He did not have any past medical history of either nephrolithiasis or exercise-induced acute renal failure to which hypouricemia sometimes predisposes [23].

We also assessed his glucose metabolism biochemically. On admission, he was treated with 25 units of insulin degludec with 1500 mg of metformin, 20 mg of teneligliptin and 20 mg of tohogliflozin with 8.9% of glycated hemoglobin (HbA1c) levels. Fasting and postprandial serum C-peptide levels-blood glucose levels were 0.09 ng/ml - 5.67 mmol/l, 1.45 ng/ml - 13.0 mmol/l, respectively while his urinary excretion of C-peptide was 25.7 μg/day, indicating impaired insulin secretion. Glutamate decarboxylase (GAD) auto-antibody was negative, and he did not have any medical histories of autoimmune diseases, excluding the possibility of autoimmune diabetes.

## Discussion and conclusions

Considering the complex web of interconnections between urate and glucose metabolism, we attempted to delineate the molecular basis behind the coprevalence of diseases observed in this case by taking advantage of whole exome sequencing. We extracted genomic DNA from his peripheral blood mononuclear cells using the QIAamp DNA Blood Maxi Kit (QIAGEN) and the sequencing library was produced by SureSelectXT Reagent Kit/SureSelectXT Human all Exon Kit V6 (Agilent Technologies). The captured DNA was sequenced using the Illumina HiSeq2500 platform with paired-end reads of 100 bp according to the manufacturer’s instructions. Data analysis was performed using the CLC Genomics Workbench (CLC Bio) and non-synonymous single nucleotide variants (SNVs) were identified following the standard workflow (Table 1). He provided written informed consent and this study was approved by the University of Tsukuba Hospital Ethics Committee with the protocol number H30–329.

**Table 1:** Summary of the whole exome sequencing in this study

	Count
Total reads	64,961,412
Mapped reads	62,114,227
Not mapped reads	2,847,185
Reads in pairs	61,126,090
Broken paired reads	988,137

**Fig. 2:**
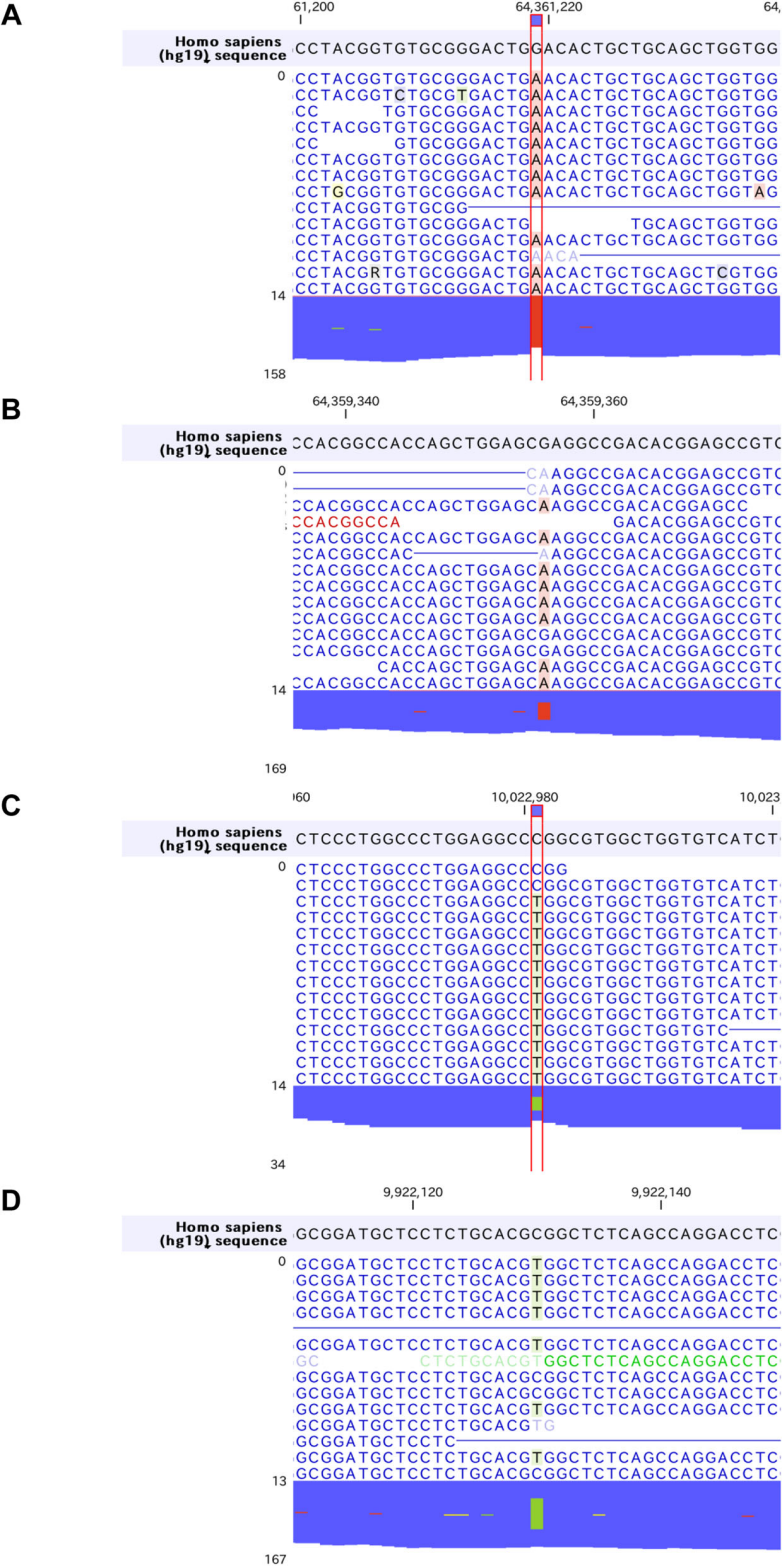
Hypouricemia associated genes. *SLC22A12* and *SLC2A9* mutations observed in this case. **a***SLC22A12* Trp258* mutation. **b***SLC22A12* Glu110Lys mutation. **c***SLC2A9* Gly25Arg mutation. **d***SLC2A9* Arg265His mutation

**Fig. 3:**
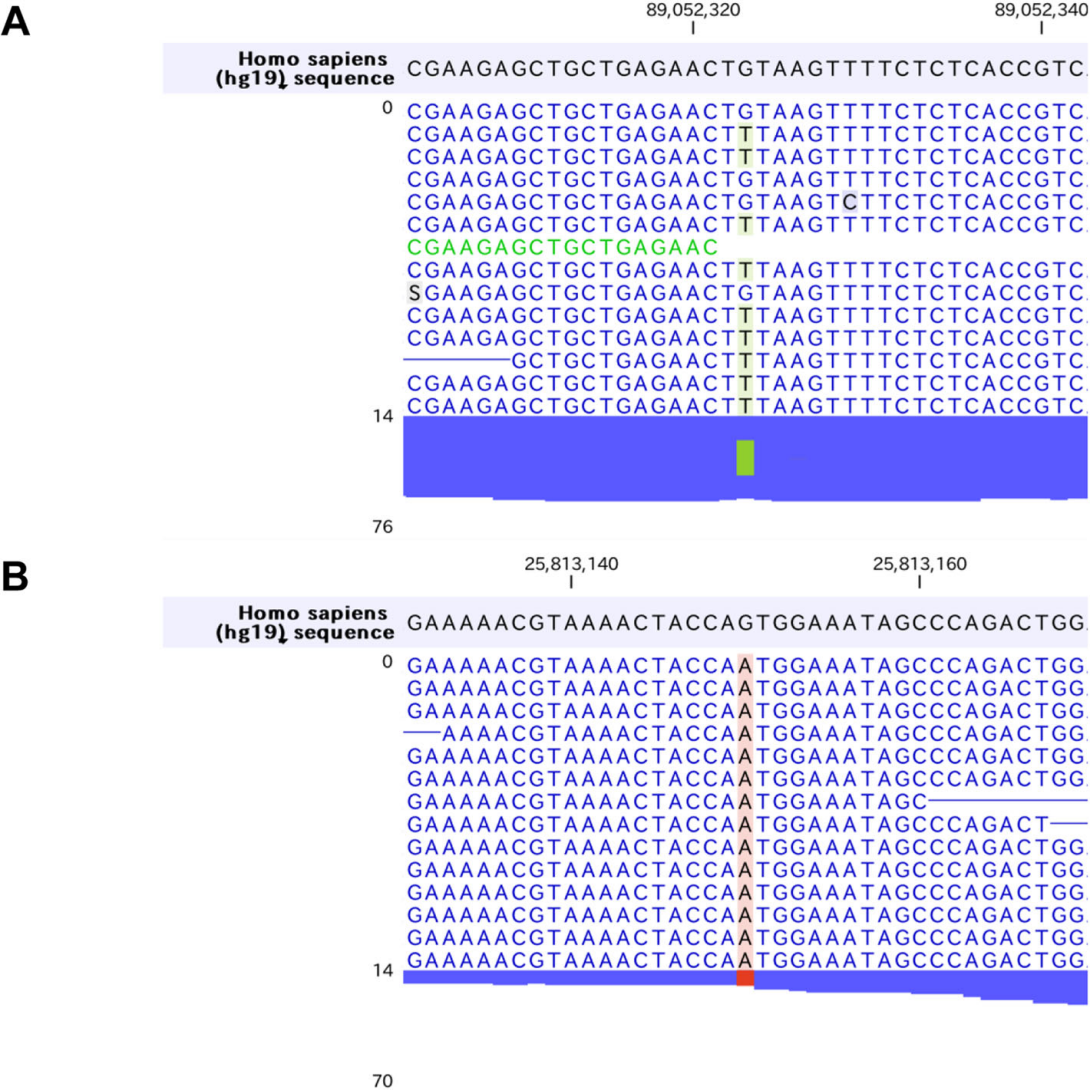
Hyperuricemia associated genes. **a***ABCG2* Gln141Lys mutation. **b***SLC17A1* Thr269Ile mutation

Firstly, we found homozygous Trp258* mutations in *SLC22A12* gene (rs121907892), that is the most commonly observed dysfunctional mutation in Japanese hypouricemic subjects (Table 2, Fig. 2a) [24, 25]. Interestingly, we additionally found novel heterozygous Glu110Lys mutation in *SLC22A12* gene (Fig. 2b). This mutation may be a C to T transition that occurred de novo, which is most frequently encountered in both the CpG and non-CpG context, typically being caused by deamination of methylated cytosines [26]. The Glu110Lys mutation would not influence the urate transport activity of SLC22A12 in this case since the SLC22A12 with Glu110Lys mutation is truncated and inactivated by the Trp258* mutation. However, considering the fact that SLC22A12 is a urate-anion exchanger [4], charged residues would be playing fundamental role in the substrate recognition or maintenance of the structural integrity. The mutation of an acidic amino acid to a basic residue would significantly alter ionic properties of SCL22A12 molecule. Whether Glu110Lys mutation on its own is sufficient to cause functional alteration of SLC22A12 and can be a risk allele for dysregulation of urate metabolism in general population awaits further investigation. On the other hand, we found two non-synonymous mutations in *SLC2A9* gene, Gly25Arg (rs2276961) and Arg265His (rs3733591), that are not causative for hypouricemia (Fig. 2c, d). Although the correlation between Arg265His mutation and hyperuricemia remains enigmatic [6, 27–31], this mutation might play a compensatory role in this hypouricemic case toward raising the serum urate levels. We further examined other genes known to be involved in urate metabolism. We found the heterozygous *ABCG2* Gln141Lys mutation (rs2231142) [9, 10, 32, 33] that has been firmly established to be associated with hyperuricemia as well as homozygous *SLC17A1* Thr269Ile mutation (rs1165196) [12, 13] also reported to be associated with hyperuricemia (Fig. 3a, b). These mutations in two genes may contribute to maintain his serum urate concentrations in the presence of hypouricemia-prone mutations (Fig. 1). Notably, ABCG2 has been reported to control serum urate levels at the level of intestine [34, 35], therefore excretion of urates into urine are increased while that into intestine may be decreased in this case. This study represents the first report showing RHUC1 gene mutations in the presence of hyperuricemia-prone gene mutations. Since the net effect of multiple gene mutations determines the serum urate levels, focused sequencing approaches for limited genes may cause some pitfalls and comprehensive cataloguing of gene mutations linked to urate levels would offer a promise to better understand the biochemical kinetics of urate metabolism in human subjects, which is critically important since urate metabolism in humans is unequivocally different from that in animal models [36]. Although nephrolithiasis and exercise-induced acute kidney failure, two major complications in RHUC, have been reported to be observed mostly in RHUC2 [8, 37], these complications can be seen in RHUC1 with *SLC22A12* Trp258* mutations as well [25]. The patient did not have any past history of these complications, which might be in part explained by the presence of the compensatory and adaptive mutations.

**Table 2:** Representative gene mutations involved in urate metabolism found in this case

Gene	Zygosity	Read count	Read coverage	Mutation	Amino acid change	SNV
*SLC22A12*	Heterozygous	49	109	G > A	Glu110Lys	
*SLC22A12*	Homozygous	129	129	G > A	Trp258*	rs121907892
*SLC2A9*	Heterozygous	19	34	C > T	Gly25Arg	rs2276961
*SLC2A9*	Heterozygous	84	190	C > T	Arg265His	rs3733591
*ABCG2*	Heterozygous	35	83	G > T	Gln141Lys	rs2231142
*SLC17A1*	Homozygous	26	26	G > A	Thr269Ile	rs1169288

**Fig. 4:**
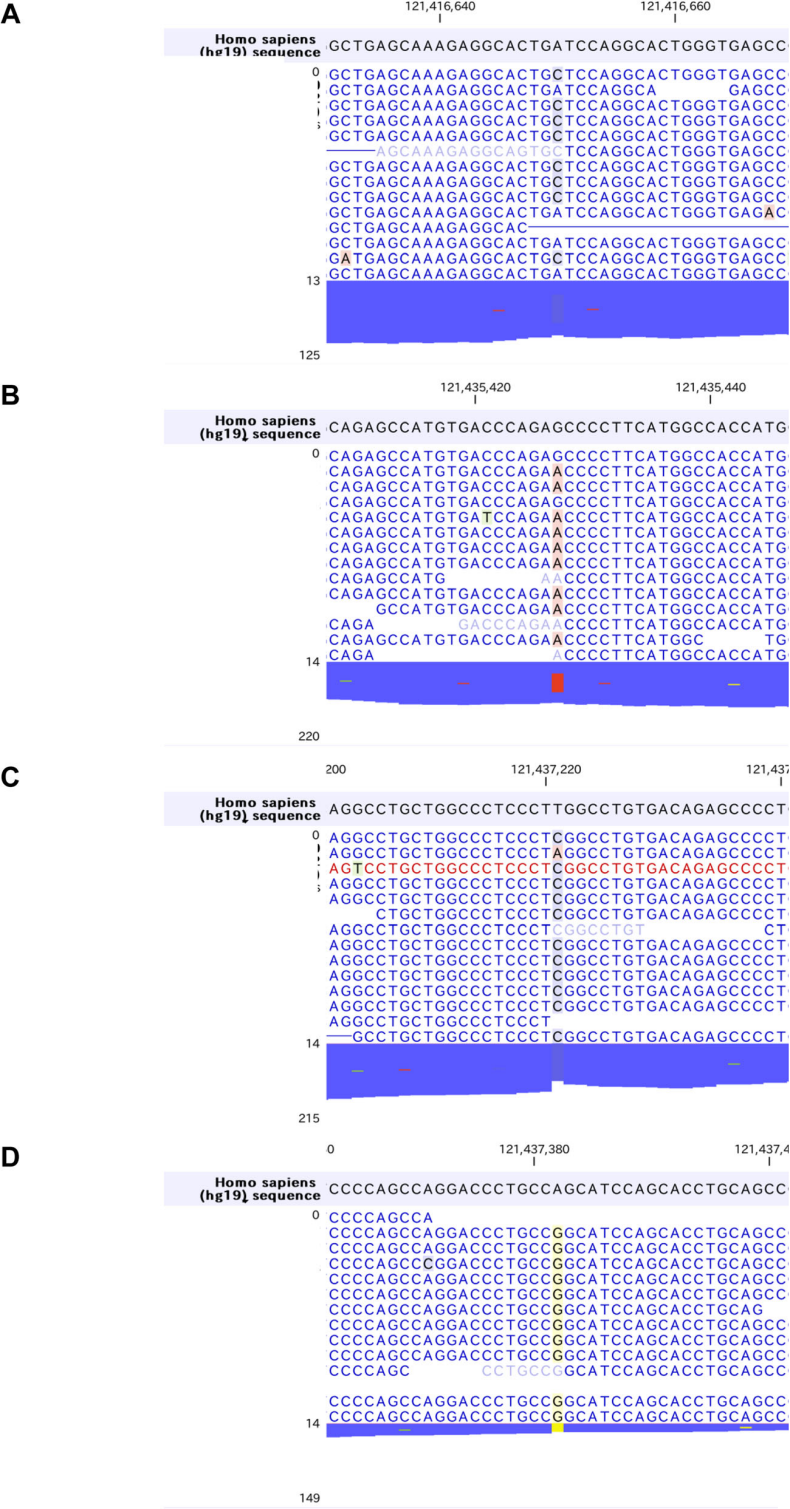
Mutations in *HNF1A* gene observed in this case. **a** Ile27Leu, **b** Ser487Asn, **c** Leu551Ser, **d** Ser581Gly

**Fig. 5:**
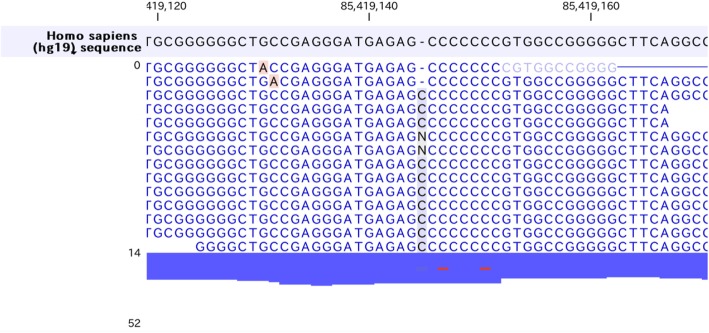
The novel mutation observed in *NKX6.1* gene

While dysfunction of SLC2A9 has been implicated in both hypouricemia [7, 8] and impaired insulin secretion [21], we could not find either dysfunctional *SCL2A9* mutations or other gene mutations causative for both of these two disorders. Therefore, we decided to search for independent gene mutations that could explain his impaired insulin secretion. Among the mutations found in genes associated with diabetes, the most promising was *HNF1A* gene (Maturity onset diabetes of the young 3, MODY3) [38, 39] where we found as many as four non-synonymous mutations accumulated in this patient: Ile27Leu (rs1169288) [40–42], Ser487Asn (rs2464196) [40, 41], Leu551Ser (rs1169304) and Ser581Gly (rs587778398) (Fig. 4a-d). Since it was reported that mutations in exons 8–10 present only in the longest isoform of *HNF1A* gene are associated with a later onset of MODY [43, 44], the latter two mutations may be of relevance to phenotypic manifestations in this case. We additionally found gene mutations in other genes associated with diabetes (Table 3) among which heterozygous Leu80fs in *NKX6.1* was an unreported mutation of potential interest (Fig. 5). The critical role of *NKX6.1* in insulin secretion from pancreatic β-cells has been demonstrated [45, 46] and this frame-shift mutation was inserted way upstream of the DNA binding domain of *NKX6.1*. Functional characterization of the mutant NKX6.1 protein and whether this can be a risk allele for diabetes in a large cohort deserve further investigation. Collectively, accumulation of these gene mutations, rather than monogenic mutations, presumably contributed to his impaired insulin secretion.

**Table 3:** Representative gene mutations associated with diabetes found in this case

Gene	Zygosity	Read count	Read coverage	Mutation	Amino acid change	SNV
*ABCC8*	Heterozygous	43	105	C > T	Val1573Ile	rs8192690
*ABCC8*	Heterozygous	33	60	C > A	Ala1369Ser	rs757110
*HNF1A*	Heterozygous	72	156	G > A	Ser487Asn	rs2464196
*HNF1A*	Homozygous	130	131	T > C	Leu551Ser	rs1169304
*HNF1A*	Homozygous	30	30	A > G	Ser581Gly	rs587778398
*HNF1A*	Heterozygous	61	125	A > C	Ile27Leu	rs1169288
*KCNJ11*	Heterozygous	31	69	C > T	Val250Ile	rs5215
*KCNJ11*	Heterozygous	50	108	T > C	Lys23Glu	rs5219
*MTNR1B*	Heterozygous	12	33	G > C	Ala107Pro	
*NKX6.1*	Heterozygous	14	42	C insertion	Leu80fs	
*PAX4*	Homozygous	31	31	T > C	*341Trp	rs712700
*PAX4*	Homozygous	13	13	T > G	His319Pro	rs712701
*PCK1*	Homozygous	53	53	G > C	Val52Leu	rs707555
*TCF7L2*	Heterozygous	73	143	C > A	His475Gln	Rs77961654
*WFS1*	Homozygous	200	200	G > A	Val333Ile	rs1801212
*WFS1*	Homozygous	153	153	G > A	Arg611His	rs734312

Analysis of genetic inheritability was very limited in this study since he had only a child alive in his family members to whom we were not able to reach. Analysis with larger sample sizes or family-based linkage analysis would help to resolve some questions raised in this study.

In conclusion, we analyzed the genome from a patient showing severe hypouricemia with diabetes with impaired insulin secretion using whole exome sequencing. Interestingly he carried hypouricemic mutations in *SLC22A12* gene as well as hyperuricemia-prone mutations. Although preceding studies have been sequencing the mutations in specific genes of interest, it would be helpful to sequence the genome in an unbiased manner to better understand urate metabolism especially from a kinetic point of view. The impaired insulin secretion may be at least in part attributed to the multiple mutations in *HNF1A* gene. We also identified novel mutations in *SLC22A12* and *NKX6.1* genes that deserve further scrutiny. This case study has instructive implications about how combined mutations in several genes affecting pathophysiology could present clinical traits in a body.

## Data Availability

The datasets generated during the current study are not publicly available because it is possible that individual privacy could be compromised.

## Abbreviations

SLC: Solute carrier family
RHUC: Renal hypouricemia
ABC: ATP-binding cassette transporter
MODY: Maturity onset diabetes of the young
BMI: Body mass index
HNF: Hepatocyte nuclear factor
NKX6.1: NK6 homeobox 1
URAT1: Urate anion transporter 1
GLUT9: Glucose transporter 9
OAT: Organic anion transporter
FEUA: Fractional excretion of urate
eGFR: Estimated glomerular filtration rate
GAD: Glutamate decarboxylase
SNV: Single nucleotide variant
